# Advances in Pulmonary Drug Delivery

**DOI:** 10.3390/pharmaceutics12100911

**Published:** 2020-09-23

**Authors:** Ayca Yıldız-Peköz, Carsten Ehrhardt

**Affiliations:** 1 Department of Pharmaceutical Technology, Faculty of Pharmacy, İstanbul University, İstanbul 34116, Turkey; aycay@istanbul.edu.tr; 2 School of Pharmacy and Pharmaceutical Sciences and Trinity Biomedical Sciences Institute, Trinity College Dublin, Dublin 2, Ireland

**Keywords:** inhaler, aerosol, lung disease, inhalation biopharmaceutics

## Abstract

Pulmonary drug delivery represents an attractive, non-invasive administration option. In addition to locally acting drugs, molecules that are intended to produce systemic effects can be delivered via the pulmonary route. Several factors need to be considered in the context of delivering drugs to or via the lungs—in addition to the drug itself, its formulation into an appropriate inhalable dosage form of sufficient stability is critical. It is also essential that this formulation is paired with a suitable inhaler device, which generates an aerosol of a particle/droplet size that ensures deposition in the desired region of the respiratory tract. Lastly, the patient’s (patho-) physiology and inhalation manoeuvre are of importance. This Special Issue brings together recent advances in the areas of inhalation device testing, aerosol formulation development, use of in vitro and in silico models in pulmonary drug deposition and drug disposition studies, and pulmonary delivery of complex drugs, such as vaccines, antibiotics and peptides, to or via the lungs.

## 1. Introduction

Drugs have been inhaled for therapeutic and recreational purposes since ancient times. The development of modern-day inhalers, e.g., pressurised metered-dose inhalers (pMDIs) and more recently, dry powder inhalers (DPIs), jet and vibrating mesh nebulisers (VMNs), and soft mist inhalers (SMIs), has given pulmonary drug delivery a momentum boost that transformed a therapeutic niche into a market predicted to hit US$41.5 billion by 2026 [1].

When delivering medicines to or via the lungs, not only the drug but also the formulation, the device and the patient need to be considered. We attempted to cover all these topics, while at the same time putting a spotlight on the up and coming areas of pulmonary drug delivery research. For this special issue, we selected 24 publications written by 131 authors hailing from 21 countries—many of whom are regular contributors to our Pulmonary Drug Delivery workshop series (www.pulmonarydrugdelivery.org). Due to the variety of subjects covered, this has become the most comprehensive special issue published by *Pharmaceutics* to date.

## 2. Devices

Adorni and colleagues studied 13 different nebulisers taking into account their aerosol output, aerosol output rate, mass median aerodynamic diameter (MMAD) and fine particle fraction according to the European Standard EN 13544-1, using sodium fluoride as a reference formulation [2]. A correlation between the aerosol quality and the nebulization rate was identified. The respirable delivered dose and respirable dose delivery rate were also determined. This study might be helpful when choosing a nebuliser depending on the drug, therapy regime and patient.

## 3. Formulations

pMDIs account for two-thirds of sold inhalers, however, due to technological advancements and environmental concerns, DPIs emerged as the preferred medical device for the treatment of a diverse range of respiratory disorders. Many DPIs contain powder mixtures of coarse carrier particles and micronised drug particles with aerodynamic particle diameters of 1–5 μm. It is estimated that only 10–15% of the drug reaches the deep lung while 20% of the drug is lost in the oropharyngeal sphere and 65% is not released from the carrier due to interparticulate adhesive forces. Lechanteur and Evrard have reviewed carrier-free particles, which are characterized by a sugar-based core encompassed by a corrugated shell layer produced by spray drying [3]. Special attention is given to the relation between the morphology (characterized by a corrugated surface) and lung deposition performance.

A different approach to overcoming the limitations of conventional carrier-based dry powders was followed by Benke et al. [4]. They report the development of an interactive physical blend of a surface-modified carrier and spray-dried meloxicam potassium with suitable shape and size for pulmonary delivery. The nonsteroidal anti-inflammatory drug was used with the intention to provide local anti-inflammatory effects to decrease the progression of cystic fibrosis (CF) and chronic obstructive pulmonary disease (COPD). In vitro and in silico studies resulted in high lung deposition, confirming that the interparticle interactions were indeed reduced in the novel formulation.

Rashid and co-workers, on the other hand, followed the traditional approach and formulated a lactose carrier-based dry powder formulation of glucagon for pulmonary delivery [5]. They investigated L-leucine and magnesium stearate as dispersibility enhancers and found the highest fine particle fraction (FPF) for the formulation to contain Mg stearate (36%) and large carrier lactose, whereas leucine was not a suitable excipient for the pulmonary delivery of glucagon.

Liquid formulation of fluticasone instead of dry powders were studied by Dogbe et al., in order to improve the biopharmaceutical performance of the drug [6]. The study compares liposomes and cyclodextrin (CyD) complexes in vitro and in vivo in mice. The in vitro tests showed no cytotoxic effects of either formulation. Fluticasone liposomes resulted in up to 30-times higher lung concentration in comparison with free drug after intranasal administration. Fluticasone hydroxypropyl-cyclodextrin complexes also showed higher lung accumulation than the free form after inhalation, however, this effect was not as pronounced as those observed with the liposomes.

## 4. Inhaled Antibiotics

Seven publications in this special issue cover various aspects of the inhalation of anti-infectives to treat lung infections. Inhaling antibiotics allows for high target site concentrations, whilst minimising systemic exposure and toxicity. Nonetheless, only a handful of antibiotics are currently marketed as nebulisable solutions or dry powders, and almost exclusively for the use in CF. Future inhaled antibiotic trials should therefore focus on disease areas outside of CF, e.g.**,** non-CF bronchiectasis, drug-resistant non-tuberculous mycobacterial infections, ventilator-associated pneumonia, post-transplant airway infections and tuberculosis (TB). Therefore, an increased number of drugs as well as novel drugs must be studied as well as other formulations.

Banaschewski and Hofmann [7] have reviewed research into completed inhaled development programs, as well as ongoing research into inhaled therapies for both non-TB mycobacterial lung disease and TB. They conclude that preclinical and clinical studies have shown that inhalation therapy, complementary to current guidance-based therapy strategies, are clinically beneficial for all types of mycobacterial infections. However, an open-minded approach should be followed to continue investigating potential additions to the antimycobacterial therapeutic arsenal.

In two papers, Sibum and colleagues report the formulation, characterisation and stability testing of high-dose dry powders of isoniazid with little or no excipient for the treatment of TB [8,9]. Initially, isoniazid was jet milled and spray dried with and without the excipient L-leucine. However, milling isoniazid did not yield a suitable formulation and spray drying the pure drug resulted in particles too large for pulmonary administration. When 5% L-leucine was added, respirable particles could be produced by spray drying but their storage stability was poor at higher relative humidity [8]. The stability was later improved by using trileucine instead of L-leucine. The optimal formulation contained 3% trileucine w/w and had a maximum fine particle dose of 58 mg when a nominal dose of 80 mg was dispersed from the Cyclops**^®^** dry powder inhaler [9].

In a case also using isoniazid, Wyszogrodzka-Gaweł et al. developed a theranostic approach to TB treatment and diagnosis that allows for imaging of the lungs by MRI [10]. Metal-organic framework (MOF) Fe-MIL-101-NH_2_ nanoparticles were loaded with isoniazid using factorial design of spray-drying with poly(lactide-co-glycolide) and leucine. The formulation thus obtained had MRI contrast capabilities, aerodynamic properties suitable for lung delivery, modified drug release and was taken up by macrophages.

Rossi and co-workers, in an attempt to treat mycobacterial lung infections, studied inhalable antibiotic powders targeting alveolar macrophages [11]. Their sodium hyaluronate-based formulation contained two antibiotics (i.e.**,** rifampicin and isoniazid) and the efflux pump inhibitor, verapamil and was produced by spray drying. The sub-micron-sized particles had a high fine particle fraction, showed a sustained release profile, were not toxic towards macrophages and achieved more than 80% reduction in bacterial viability in susceptible and resistant *M*. *tuberculosis* strains in vitro.

Isoniazid was introduced in 1952. Bedaquiline, on the other hand is a relatively novel oral anti-TB drug that was approved in the US in 2012 by fast-track accelerated approval and is on the World Health Organization’s List of Essential Medicines. Bedaquiline, however, has a black-box warning of increased risk of death and arrhythmias. Hence, Momin et al. developed inhalable bedaquiline dry powder particles with the intention of reducing the systemic side-effects [12]. Bedaquiline was processed by spray drying and the resulting microparticles were stable during one-month of storage. Spray-dried bedaquiline was non-toxic in respiratory epithelial cell cultures and effectively inhibited the growth of *M. tuberculosis* in vitro.

Antimicrobial peptides (AMPs) are being considered as alternatives to conventional antibiotics. AMPs do not only have direct antimicrobial activity, but also modulate the immune system and wound repair, making them of interest in CF therapy. Forde and colleagues studied whether prodrugs of AMPs (pro-AMPs) can be delivered by VMN and whether modifications of pro-AMP had an effect on the delivery [13]. Nebulisation did not alter AMPs’ physical characteristics and antimicrobial activity. Approximately 25% of the nominal dose was delivered in a spontaneous breathing setting, with higher delivery rates observed in a mechanically-ventilated model. These results demonstrated the feasibility of AMP delivery using a VMN and also that the prodrug modification is not detrimental.

## 5. Pulmonary Delivery of Biopharmaceuticals

Vaccines against bacterial diseases may directly reduce antibiotic use through reduction of disease incidence. Thus, immunisation has the potential to reduce antibiotic use. Vaccine delivery via mucosal surfaces is an interesting alternative to parenteral vaccination and in many cases resembles the route taken by the microorganism when entering the body. Hellfritsch and Scherließ in their review provide an introduction to respiratory vaccination, formulation approaches and application strategies [14].

## 6. Inhaled Anti-Cancer Treatment

Another disease that could benefit from the advantages that inhalation therapy offers in terms of reduced systemic drug burden is lung cancer. Parvathaneni et al. in their study investigated the anti-tumour effects of liposomally-encapsulated pirfenidone in vitro [15]. Pirfenidone, a repurposed anti-fibrotic drug, was encapsulated in cationic liposomes. The formulation was successfully aerosolised by a jet nebuliser and showed promising anti-tumour effects in various human lung cell lines compared to free pirfenidone.

## 7. In Vitro and In Silico Deposition and Drug Disposition

Particle deposition in the lungs is associated with the breathing patterns of the patient and also pathophysiological changes due to lung diseases. In their study, Farkas and colleagues measured realistic inhalation profiles of mild, moderate, and severe COPD patients and simulated the deposition patterns of the Symbicort^®^ Turbuhaler^®^ in comparison to data generated from healthy control subjects [16]. They found an association between the amount of drug deposited within the lungs and disease severity. The results from this study suggest that to receive a similar lung concentration, severe COPD patients would require much higher doses than healthy individuals.

Tailoring the shape and size of fibre-like aerosols to achieve targeted pulmonary drug delivery with increased deposition efficiency is an interesting concept. Shachar-Berman et al. calculated the transport and deposition characteristics of fibres under physiological inhalation conditions in silico using computational fluid dynamics (CFD) simulations [17]. Aerosol deposition was quantified as a function of the equivalent diameter (dp) and geometrical aspect ratio (AR). They found that high AR fibres in the narrow range of dp = 6–7 µm mainly deposited in the upper airways, whereas fibres in the range of dp = 4–6 µm penetrated all the way to distal lung regions.

To prolong the duration of the effect in the lungs, increasing the drug’s affinity to lung tissue is an important strategy for drug development. However, differences in lung structure and blood flow affect local pulmonary drug disposition. Himstedt and co-workers studied regional lung distribution of four drugs (i.e.**,** salmeterol, fluticasone propionate, linezolid and indomethacin) after intravenous administration in rats [18]. In addition, a semi-mechanistic model was employed to describe the observed tissue drug concentrations. The in silico model was able to explain the pulmonary pharmacokinetics of the two neutral and one basic model drug based on their tissue specific affinities (Kp) and organ blood flow. The pulmonary PK of indomethacin, however, could not be modelled, suggesting that acidic drugs have different pulmonary PK characteristics.

In their paper, Salomon et al. studied the activity of carnitine transporter OCTN2, which is associated with asthma and other inflammatory lung diseases. They studied freshly isolated human alveolar type I (ATI)-like epithelial cells in primary culture and several respiratory epithelial cell models [19]. [^3^H]-acetyl-l-carnitine uptake and pharmacological inhibition was determined in ATI-like, NCl-H441, A549 and Calu-3 cells. It was concluded that OCTN2 is involved in the cellular uptake of acetyl-l-carnitine at the alveolar epithelium, however none of the tested cell lines are optimal surrogates for primary cells in carnitine transport studies.

## 8. Environmental Exposure and Toxicity Studies

Pulmonary drug delivery research is usually mainly concerned with administering aerosols to the lungs. The non-deposited, exhaled dose, however, can be a significant health hazard in both clinical and homecare settings. In two publications, McGrath and colleagues used nebulised albuterol sulphate solution when they investigated fugitive aerosol emissions from two commercially available nebulisers in combination with an open or valved facemask or using a mouthpiece with and without a filter [20] and during high flow nasal cannula (HFNC, see below for more on HFNC) therapy [21], respectively. It was shown that the MMAD of the fugitively-emitted aerosols was less than 1 µm, while the initially generated aerosols were between 2 and 5 µm. A facemask combination resulted in the highest time-averaged fugitively-emitted aerosol concentrations, whereas a filter on the exhalation port of the mouthpiece yielded the lowest concentrations. In the HFNC study, fugitive aerosol emissions were influenced by the interface type, patient and supplemental gas-flow rate, with fugitive aerosol MMAD decreasing with an increasing flow rate. These findings are important in developing policy and best practice for risk mitigation from fugitive emissions.

‘Foamy’ alveolar macrophages (FAM) may be indicators of drug-induced phospholipidosis. Currently, orally administered amiodarone is used to induce pulmonary phospholipidosis. Patel et al. in their study investigated if pulmonary delivery of amiodarone in rats could be established as a novel phospholipidosis-induced FAM model in comparative inhalation toxicology [22]. A high dose of aerosolised amiodarone caused transient pulmonary inflammation, however, only oral delivery resulted in FAM.

## 9. High Flow Nasal Cannula Therapy

High flow nasal cannula (HFNC) is widely utilized to support critically ill adults, paediatrics and neonates. Through the continuous delivery of oxygen at high flow rates that meet or exceed patients’ inspiratory flow, HFNC improves oxygenation, respiratory rates, patient comfort, and tolerance during therapy. As HFNC becomes more widely employed, the technique is also being considered for aerosol drug delivery. Ji et al. have identified the ratio of nasal cannula gas flow to patient inspiratory flow as a primary independent predictor of inhaled dose. When the ratio was <1, the inhaled dose was higher than those with ratio > 1. The inhaled dose was also more consistent with quiet and distressed breathing with ratio < 1 [23]. In a separate study, Alcoforado and co-workers observed that both flow and active heated humidity inversely impacted aerosol delivery through HFNC. Nonetheless, aerosol administration across the range of commonly used flows can provide measurable levels of lung drug deposition in healthy adult subjects [24]. Ji and colleagues retrospectively analysed study data on HFNC-delivery epoprostenol (iEPO), utilised to improve oxygenation in mechanically ventilated patients with severe hypoxemia comorbid with pulmonary hypertension or right heart dysfunction [25]. Their data suggest that iEPO via HFNC can improve oxygenation in adult patients and supports the need for a larger prospective randomised control trial to further evaluate the efficacy of iEPO via HFNC.

## 10. Conclusions

In this Special Issue, a cross-section of current research in the field of pulmonary drug delivery is published. There is still a lot of work to be done in the areas of inhaler devices and formulation development, particularly, with regards to dry powder and colloidal systems. A severe limitation in this field of research is the small number of excipients FDA/EMA-approved for inhalation.

Topical delivery of antibiotics appears to be an area that has attracted a lot of interest in recent years and is likely to make an even bigger impact in the treatment of pulmonary infections in the future. Moreover, viral lung diseases such as COVID-19 are a challenge and delivering antivirals by inhalation might be an approach worth considering.

In addition to infectious diseases, conditions such as lung cancer are being actively researched in the context of inhalation drug delivery. The respiratory route can also be utilised to achieve mucosal vaccination against bacterial or viral infections.

Furthermore, there is a tangible shift from lab-based experiments towards in silico studies, e.g., in the areas of deposition modelling and physiology-based pharmacokinetic modelling. In the foreseeable future, however, the computer-based approached will need to be based on real-life data generated in actual experiments in the lab or the clinical setting. In the context of data generation, scientists should focus on novel techniques to study the fate of inhaled drugs, in order to allow in vivo/in vitro correlations and predictions.
